# Conidial Melanin of the Human-Pathogenic Fungus Aspergillus fumigatus Disrupts Cell Autonomous Defenses in Amoebae

**DOI:** 10.1128/mBio.00862-20

**Published:** 2020-05-26

**Authors:** Iuliia Ferling, Joe Dan Dunn, Alexander Ferling, Thierry Soldati, Falk Hillmann

**Affiliations:** a Junior Research Group Evolution of Microbial Interactions, Leibniz Institute for Natural Product Research and Infection Biology—Hans Knöll Institute (HKI), Jena, Germany; b Institute of Microbiology, Friedrich Schiller University Jena, Jena, Germany; c Department of Biochemistry, Faculty of Science, University of Geneva, Geneva, Switzerland; d Heid-Tech, Technische Schule Heidenheim, Heidenheim an der Brenz, Germany; Universidade de Sao Paulo

**Keywords:** *Aspergillus fumigatus*, *Dictyostelium discoideum*, *Protostelium aurantium*, DHN-melanin, phagocytosis, phagosome maturation, acidification, V-ATPase, ROS, NADPH oxidase, membrane damage, membrane repair, ESCRT machinery, fungi, amoeba, pathogens

## Abstract

The human-pathogenic fungus Aspergillus fumigatus is a ubiquitous saprophyte that causes fatal lung infections in immunocompromised individuals. Following inhalation, conidia are ingested by innate immune cells and can arrest phagolysosome maturation. How this virulence trait could have been selected for in natural environments is unknown. Here, we found that surface exposure of the green pigment 1,8-dihydroxynaphthalene-(DHN)-melanin can protect conidia from phagocytic uptake and intracellular killing by the fungivorous amoeba Protostelium aurantium and delays its exocytosis from the nonfungivorous species Dictyostelium discoideum. To elucidate the antiphagocytic properties of the surface pigment, we followed the antagonistic interactions of A. fumigatus conidia with the amoebae in real time. For both amoebae, conidia covered with DHN-melanin were internalized at far lower rates than were seen with conidia lacking the pigment, despite high rates of initial attachment to nonkilling D. discoideum. When ingested by D. discoideum, the formation of nascent phagosomes was followed by transient acidification of phagolysosomes, their subsequent neutralization, and, finally, exocytosis of the conidia. While the cycle was completed in less than 1 h for unpigmented conidia, the process was significantly prolonged for conidia covered with DHN-melanin, leading to an extended intracellular residence time. At later stages of this cellular infection, pigmented conidia induced enhanced damage to phagolysosomes and infected amoebae failed to recruit the ESCRT (endosomal sorting complex required for transport) membrane repair machinery or the canonical autophagy pathway to defend against the pathogen, thus promoting prolonged intracellular persistence in the host cell and the establishment of a germination niche in this environmental phagocyte.

## Abstract (precis)

Infections with Aspergillus fumigatus are usually acquired by an inhalation of spores from environmental sources. How spores of a saprophytic fungus have acquired abilities to withstand and escape the phagocytic attacks of innate immune cells is not understood. The fungal surface pigment dihydroxynaphtalene-melanin has been shown to be a crucial factor for the delay in phagosome maturation. Here, we show that this pigment also has a protective function against environmental phagocytes. Pigmented conidia escaped uptake and killing by the fungus-eating amoeba Protostelium aurantium. When ingested by the nonfungivorous phagocyte Dictyostelium discoideum, the pigment attenuated the launch of cell autonomous defenses against the fungal invader, such as membrane repair and autophagy, leading to prolonged intracellular retention. Membrane damage and cytoplasmic leakage may result in an influx of nutrients and thus may further promote intracellular germination of the fungus, indicating that A. fumigatus has acquired some of the basic properties of intracellular pathogens.

## INTRODUCTION

The ubiquitous filamentous fungus Aspergillus fumigatus is most commonly found in the soil or on decaying organic matter and infects immunocompromised individuals after inhalation of conidia. Worldwide, over 200,000 life-threatening infections are caused by A. fumigatus annually, with mortality rates of infected individuals ranging from 30 to 90% (1, 2). Poor diagnosis, often-rapid disease progression, and gaps in our understanding of the early stages of infection are currently limiting therapeutic options.

The green-grayish conidial pigment 1,8-dihydroxynaphthalene (DHN)-melanin is among the first microbe-associated molecular patterns that the host encounters during infection. Myeloid and endothelial cells of the lung recognize DHN-melanin itself via the C-type lectin receptor MelLec that plays an important role in the protective antifungal immunity of both mice and humans (3). It has been shown that the presence of DHN-melanin interferes with conidial uptake and processing in mammalian phagocytes and can inhibit apoptosis in endothelial lung cells (4–10). Recent experiments with macrophages showed that melanized conidia of A. fumigatus interfere with phagosome acidification by preventing the formation of lipid rafts that are essential for V-ATPase proton pump assembly (11). Upon germination, the DHN-melanin layer is lost, exposing chitin, glycoproteins, and β-1,3-glucan, exposure of which facilitates recognition, phagocytic uptake, and killing by immune cells (12, 13). The biochemical fate of fungal melanin following swelling and germination of the conidia is thus far unknown.

In contrast to commensal fungal pathogens such as Candida albicans, A. fumigatus is considered an environmentally acquired pathogen, as it is frequently isolated from natural reservoirs and occupies a well-established niche as a decomposer of organic matter. During evolution, it can thus be expected that A. fumigatus has acquired counterdefense strategies against phagocytic predators that might explain the virulence of environmental pathogens for humans. This hypothesis was recently named the “amoeboid predator-animal virulence hypothesis” (14). According to this hypothesis, microorganisms have trained their virulence through competition with microbial predators. Undermining this hypothesis, A. fumigatus conidia can either be killed by the fungivorous amoeba Protostelium aurantium or survive and germinate in other soil amoeba such as Acanthamoeba castellanii or Dictyostelium discoideum (15–18).

While P. aurantium can actively feed on fungi, including A. fumigatus, D. discoideum feeds primarily on bacteria and protects itself from potentially harmful intracellular pathogens via cell autonomous defenses such as a microbicidal phagolysosome (PL), the ESCRT membrane repair machinery, or activation of autophagy (19–21). To avoid infection, the phagocytic host can eliminate pathogens by forming a plasma membrane-derived phagosome before they can escape or establish a survival niche. By a sequence of membrane fusion and fission events, known as phagosome maturation, the phagosome acquires its full range of microbicidal and degradative features (22). The final step of phagosome maturation is its resolution, during which the phagosomal content becomes recycled, and indigestible material is exocytosed (see reference 20 for review). Such a constitutive exocytosis is exploited by D. discoideum upon infection with the intracellular fungal pathogen Cryptococcus neoformans (23). In turn, the rate of formation of the virulence factor l-3,4-dihydroxyphenylalanine (l-DOPA)-melanin was found to be accelerated in C. neoformans after passage through D. discoideum as a host (24).

As DHN-melanin of A. fumigatus was shown to serve a protective role during interactions with phagocytes from mammalian hosts, we have used the two model amoebae, P. aurantium and D. discoideum, to assess whether the pigment of the fungus has an even broader impact on various environmental phagocytic hosts and thereby provides a selective advantage for the fungus in its natural environment.

## RESULTS

### Conidia covered by 1,8-DHN-melanin escape ingestion and killing by the fungivorous amoeba Protostelium aurantium.

**FIG 1:**
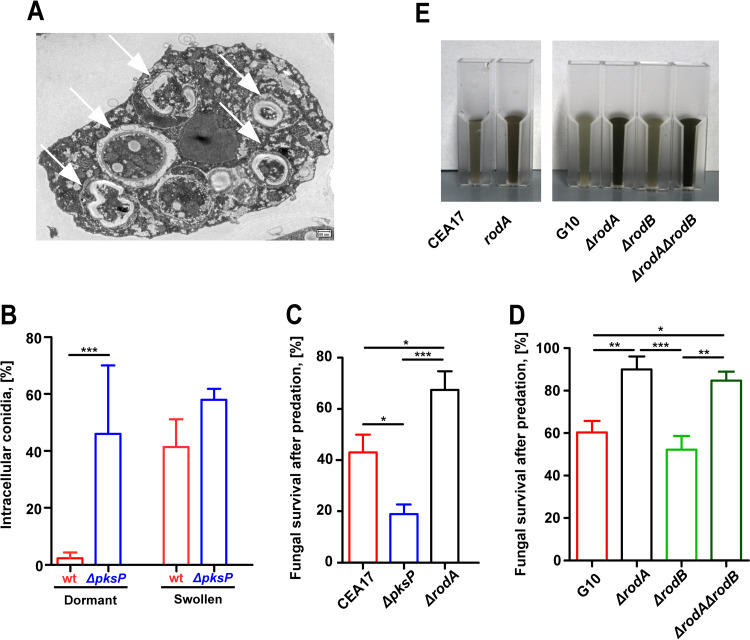
Phagocytosis and killing of Aspergillus fumigatus by Protostelium aurantium. (A) Transmission electron micrograph (TEM) of P. aurantium with internalized conidia of A. fumigatus (white arrows) at different stages of digestion. (B) Uptake of dormant or swollen spores of A. fumigatus conidia after 1 h of coincubation by P. aurantium. wt, wild type. (C and D) Viability of conidia after P. aurantium predation. Fungal survival of the CEA17, Δ*pksP*, and Δ*rodA* strains (C) and the G10, Δ*rodA*, Δ*rodB*, and Δ*rodA* Δ*rodB* strains (D) was determined from resazurin-based measurements of fungal growth following confrontations with P. aurantium at an MOI of 10. Fungal survival data are expressed as means and standard errors of the means (SEM) of results from three independent experiments. Statistically significant differences were calculated with a Bonferroni posttest after a two-way ANOVA, with significance shown as follows: *, *P* < 0.05; **, *P* < 0.01; ***, *P* < 0.001. (E) Suspensions of 10^9^ conidia of A. fumigatus strains showing different levels of DHN-melanin exposure. CEA17 and G10 represent wild type-like strains; Δ*pksP* is a deletion mutant for the first gene in 1,8-DHN-melanin biosynthesis; Δ*rodA*, Δ*rodB*, and Δ*rodA* Δ*rodB* are deletion mutants for genes encoding surface hydrophobins.

Protostelium aurantium is a specialized mycophagous predator able to feed on yeast cells, invade fungal hyphae, and ingest fungal conidia that have initiated swelling as an onset of their germination (18). Internalized conidia of A. fumigatus eventually undergo intracellular digestion (Fig. 1A). When confronted with the fungivorous amoeba Protostelium aurantium, resting, melanin-deficient conidia lacking the gene for DHN biosynthesis (*ΔpksP*) were ingested at far higher levels than resting, wild-type conidia covered with the green-grayish pigment. This difference was not seen for swollen conidia, as these are generally devoid of DHN-melanin, and high phagocytic efficiencies were seen for swollen conidia of both strains (Fig. 1B). Enhanced uptake of the white *ΔpksP* conidia also corresponded to more effective killing than was seen with the greenish pigmented wild-type conidia (Fig. 1C). In line with the comparably higher exposure of DHN-melanin on the surface of conidia lacking the gene encoding the main conidial hydrophobin RodA (25), the rate of survival of these RodA-deficient fungal strains was even higher than that of wild-type conidia (Fig. 1D and E). These findings demonstrate that a DHN-melanin-covered cell surface can provide protection against this fungivorous predator.

### Conidia covered by 1,8-DHN-melanin are ingested less frequently by D. discoideum.

**FIG 2:**
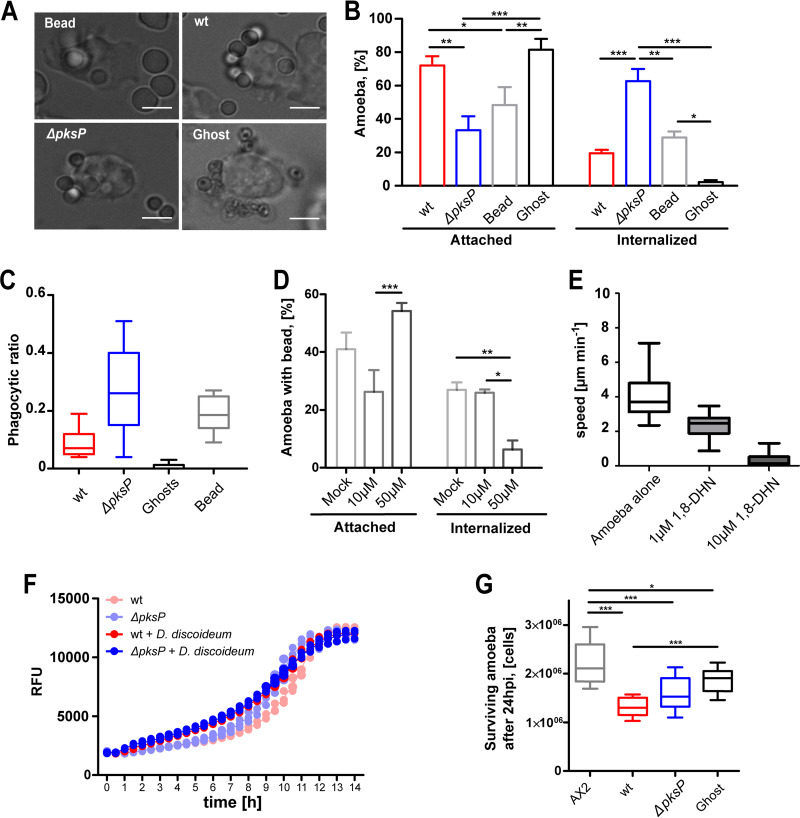
Phagocytosis of Aspergillus fumigatus conidia by Dictyostelium discoideum. (A) Resting conidia of the A. fumigatus wild-type strain (wt) or the melanin-deficient strain (Δ*pksP*) were added to D. discoideum at an MOI of 5. Silica beads (Bead) and melanin ghosts (Ghost) were added to the amoebae at the same MOI. Images were captured after 1 h of coincubation. The scale bars are 5 μm. (B) Cells with events of attachment or phagocytosis were quantified from images captured at 1 h p.i. The bars represent means and SEM of results from three independent experiments, with *n* = 100 for each experiment. Statistically significant differences were calculated with a Bonferroni posttest after a two-way ANOVA, with asterisks indicating significance (*, *P* < 0.05; **, *P* < 0.01; ***, *P* < 0.001). (C) Phagocytic ratio for A. fumigatus conidia, silica beads, and melanin ghosts. (D) Amoebae were exposed to silicon beads in the presence of 10 or 50 μM 1,8-DHN. Imaging and quantification were carried out as described for panel B. (E) Velocity of D. discoideum immediately after addition of 1,8-DHN to the medium. (F) Resazurin-based measurement of fungal survival after coincubation with D. discoideum at an MOI of 0.01. RFU, relative fluorescence units. (G) Viable amoeba cells after 24 h of incubation with the fungus. Statistically significant differences were calculated with a Bonferroni posttest after two-way ANOVA (*, *P* < 0.05; **, *P* < 0.01; ***, *P* < 0.001).

Prior to phagocytic uptake, host receptors generally engage with ligands exposed on the surface of A. fumigatus conidia. This association with its ligand initiates signaling pathways that cause the extension of lamellipodia, which surround the particle and generate the nascent phagosome. To test whether DHN-melanin could also inhibit uptake, wild-type and *pksP* mutant conidia of A. fumigatus were confronted with the model amoeba D. discoideum. After 1 h of coincubation, we found that D. discoideum amoebae had ingested 63% of the melanin-deficient *ΔpksP* conidia but only 20% of the wild-type conidia (Fig. 2A and B). The phagocytic efficiencies determined for wild-type and *ΔpksP* conidia were lower and higher than those determined for inert silica particles, respectively (Fig. 2C; see also Fig. S1 in the supplemental material). Melanin ghosts (i.e., empty shells of melanin) obtained after harsh chemical treatment of wild-type conidia were rarely taken up by the amoeba. However, these melanin ghosts were found to associate readily with the amoeba cell wall, covering the entire surface (Fig. 2A and B; see also Fig. S1). Collectively, our results suggested that DHN-melanin might have an impact on the uptake and intracellular processing of conidia. This supposition was further supported by an experiment performed with the DHN-melanin monomer 1,8-dihydroxynaphthalene, which repressed the phagocytosis of beads and the motility of the amoeba in a dose-dependent manner (Fig. 2D and E).

10.1128/mBio.00862-20.1 FIG S1 Efficiency of phagocytosis of resting conidia of A. fumigatus by D. discoideum illustrated as the uptake ratio φ_a_ (amoeba perspective). N_a_^Phag^, number of amoebae with at least one ingested particle; N_a,_ total number of amoebae. Download FIG S1, TIF file, 0.05 MB. Copyright © 2020 Ferling et al. 2020 Ferling et al. This content is distributed under the terms of the Creative Commons Attribution 4.0 International license.

Although deformed or degraded fungal conidia after coincubation of swollen spores with D. discoideum were occasionally observed after confrontation for 3 to 5 h (Fig. S2A and B), the fungal viability of either the wild type or the melanin-deficient mutant was not markedly altered even at a multiplicity of infection (MOI) of 0.01 (1 conidium in 100 amoebae cells) (Fig. 2F). In turn, the viability of D. discoideum was significantly affected after confrontation with the fungus at an MOI of 0.1 (Fig. 2G). These effects were largely independent of DHN-melanin.

10.1128/mBio.00862-20.2 FIG S2 Viability of swollen conidia of A. fumigatus and D. discoideum after the confrontation. TEM (A) and light microscopy (B) images show D. discoideum containing swollen conidia or conidia-like inclusions at different stages of digestion. Download FIG S2, TIF file, 0.1 MB. Copyright © 2020 Ferling et al. 2020 Ferling et al. This content is distributed under the terms of the Creative Commons Attribution 4.0 International license.

### 1,8-DHN-melanin delays the maturation of phagolysosomes and mediates prolonged intracellular transit.

**FIG 3:**
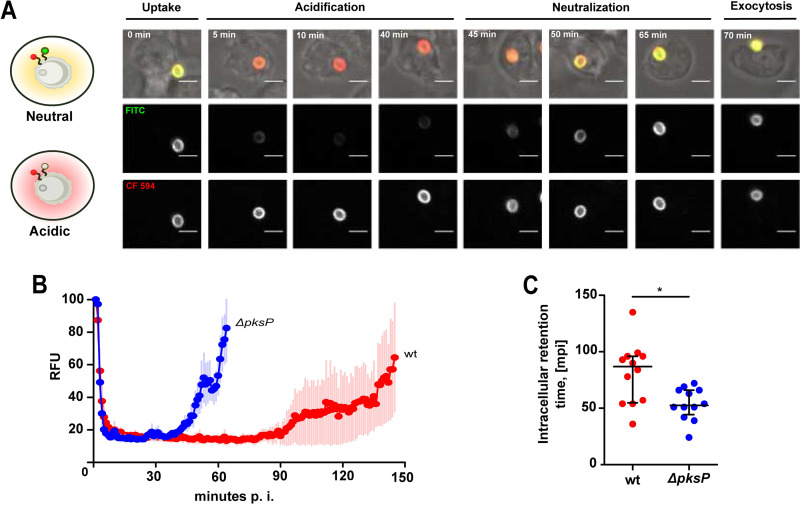
Acidification and intracellular transit of conidia in D. discoideum. Amoebae were infected with resting conidia of the wild-type strain or the Δ*pksP* strain prestained with a pH-sensitive fluorophore (FITC) and the reference fluorophore (CF594) for real-time measurements of acidification and residence time in the amoeba. (A) Time-lapse illustration of major steps during the phagocytic cycle for resting conidia of the Δ*pksP* mutant. (B) Timeline of FITC-derived fluorescence intensity indicating pH variations at the conidial surface during phagocytosis. (C) Intracellular retention time of conidia inside D. discoideum. Statistically significant differences were calculated with a *t* test.

We were further interested in the intracellular fate of the conidia after ingestion by a nonfungivorous phagocyte and thus followed the infection process at the single-cell level. To monitor the intracellular transit of conidia in D. discoideum, conidia were stained with fluorescein isothiocyanate (FITC) (green) and with CF594 (red) for the normalization of signal intensity (Fig. 3A; see also Fig. S3A). Ratiometric calculations of the differences between the two dyes, with FITC responding to changes in pH, allowed us to track the phagosomal pH dynamics for conidia over the entire intracellular period (Fig. 3B; see also Fig. S3B to D). These measurements demonstrated that both wild-type and *ΔpksP* mutant conidia underwent rapid acidification followed by neutralization and subsequent exocytosis. The initial acidification occurred within 5 min of uptake for both strains (dashed lines) and thus at a rate too high to delineate any significant differences. This sequence in phagosomal processing was previously also observed for inert particles completing the process within 40 min to 1 h (23, 26). Phagosome maturation and exocytosis of resting, melanin-deficient conidia were completed on a time scale similar to that seen with inert particles (50 min). However, wild-type conidia resided significantly longer inside the phagolysosome (PL) (90 min in average; Fig. 3C). It is also noteworthy that for wild-type conidia, phagosomal neutralization was comparably slow and the intraphagosomal pH was highly variable at later stages, suggesting interference at single or multiple stages of the phagosome maturation process. Our results show that the structure of a conidium-containing phagosome in D. discoideum is highly dynamic. The different rates for uptake, acidification, and intracellular transit were integrated into a Monte Carlo simulation, predicting that in long-term confrontations, conidia can be found at variable stages of phagolyosomal maturation (Fig. S4).

10.1128/mBio.00862-20.3 FIG S3 FITC-based pH measurement for conidia of A. fumigatus. (A) Time-lapse illustration of major steps during the phagocytic cycle for resting conidia of the wild type prestained with the pH-sensitive fluorophore (FITC) and the reference fluorophore (CF594) to measure acidification and retention time of conidia in D. discoideum. The scale bar is 5 μm. (B) Example of a calibration curve for the FITC-stained fungal conidia imaged at defined pH values. (C) For the determination of fluorescence intensities, 10 random conidia were imaged in media buffered at different pH levels. (D) The internalized fungal conidia resided in phagosomes with low pH and assembled VatB-RFP on the surface. Download FIG S3, TIF file, 0.7 MB. Copyright © 2020 Ferling et al. 2020 Ferling et al. This content is distributed under the terms of the Creative Commons Attribution 4.0 International license.

10.1128/mBio.00862-20.4 FIG S4 Monte Carlo prediction of the phagolysosomal acidification states throughout the amoeba population. (A) Schematic setup for a computational simulation of population dynamics based on experimentally determined parameters in single-cell analyses. (B) Percentages of acidic phagosomes in the population of 10^4^ amoebae infected with wild-type and Δ*pksp* conidia at an MOI of 10. Parameters included from experimental data for wild-type and *ΔpksP* conidia, respectively, are as follows: conidial uptake probability of 20 and 63%, acidic time spans of 52 and 32 min, and exocytosis at 87 and 53 min after uptake. Infections with melanized versus nonmelanized conidia yielded 100% versus 82% of acidified phagolysosomes after 30 min p.i., respectively, while 36% of phagolysosomes containing wild-type conidia and 48% of those containing *ΔpksP* conidia were acidified after 5 h of coincubation. As the period between uptake and acidification of the phagosome was too short to allow accurate measurement, we set this value to zero, which may have influenced the precision of the computational model. (C and D) Experimentally determined trafficking of the V-ATPase at 30 min p.i. (C) and 300 min p.i. (D) confirmed the Monte Carlo model prediction with reasonable accuracy. Statistically significant differences were calculated with a Bonferroni *post hoc* test after a two-way ANOVA (*P* < 0.0001). Download FIG S4, TIF file, 0.2 MB. Copyright © 2020 Ferling et al. 2020 Ferling et al. This content is distributed under the terms of the Creative Commons Attribution 4.0 International license.

### Functional V-ATPase is trafficked to A. fumigatus-containing phagosomes.

The initial acidification occurred within 5 min of ingestion and was largely independent of the presence of DHN-melanin. To visualize the real-time distribution of the proton-pumping enzyme complex, we employed D. discoideum strains expressing fusions of the V-ATPase membrane channel subunit VatM and the cytosolic domain VatB with green fluorescent protein (GFP) and red fluorescent protein (RFP), respectively (27, 28).

**FIG 4:**
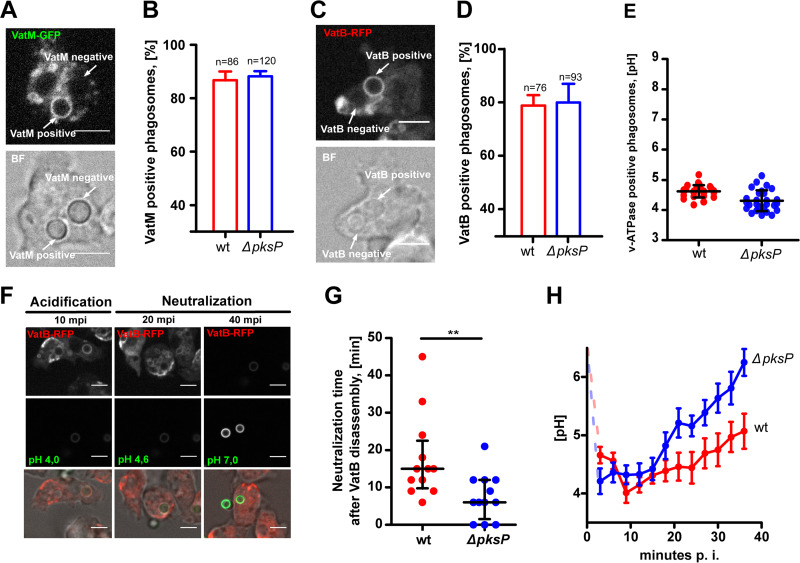
V-ATPase assembly and phagosomal acidification during conidial infection. (A and C) Representative images of VatM-GFP (A) and VatB-RFP (C) localization after 30 min p.i. The scale bars are 5 μm. BF, Brightfield. (B and D) Percentages of VatM-GFP-positive (B) and VatB-RFP-positive (D) phagosomes after 30 min p.i. Experiments were performed in 3 biological replicates. (E) Measurements of the pH inside V-ATPase-positive phagosome were carried out at different stages of infection. Analyses were performed in three independent experiments, and bars indicate means and SEM. (F) Representative images of different stages of phagosome maturation in VatB-RFP-expressing cells infected with FITC-stained Δ*pksP* conidia. Scale bar is 5 μm. (G) Phagosomal neutralization time (pH 6.0) after VatB disassembly from the phagosomal surface. Statistically significant differences were calculated with a Student's *t* test with *P* = 0.0066. (H) Kinetics of the phagosomal pH in VatB-RFP-expressing cells infected with resting conidia. Twelve independent movies were taken for each fungal strain. Dots and error bars indicate means and standard errors (SE), respectively.

Live, single-cell imaging of FITC-stained conidia after uptake by VatB-RFP-expressing amoeba demonstrated that VatM and VatB were trafficked to conidium-containing phagolysomes within the first 30 min of the interaction (Fig. 4A to D). Trafficking of the V-ATPase fully correlated with PL acidification for both types of conidia (Fig. 4E). Fast acidification and V-ATPase trafficking to the phagosomal membrane was followed by its retrieval and subsequent neutralization of the phagosomal lumen, with conidial exocytosis as the final step (Fig. 4F). As expected from the previous experiments, the neutralization kinetics for wild-type and *ΔpksP* conidia varied significantly. While both fungal strains triggered maximum acidification within less than 10 min, amoebae infected with wild-type conidia were delayed in reaching the maximum pH (Fig. 4G). Also, following VatB-RFP retrieval, the phagosomes took significantly longer to reach pH 6 again when infected with DHN-melanin-covered wild-type conidia than the *ΔpksP* conidium-containing phagosomes (Fig. 4H).

### ROS generation coincides with neutralization of A. fumigatus-containing phagolysosomes.

**FIG 5:**
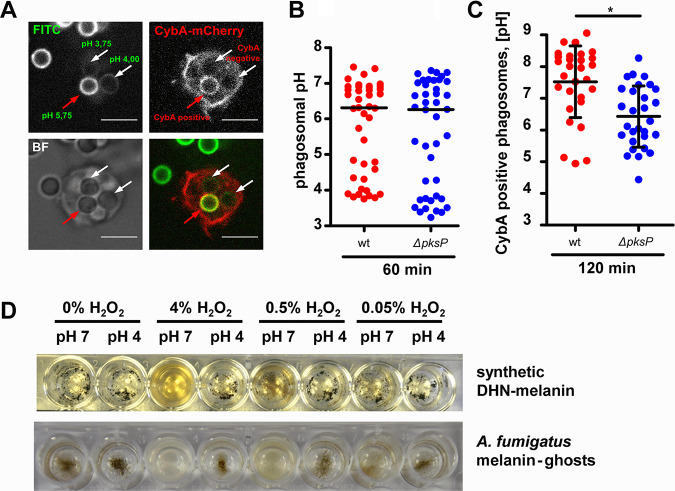
NADPH oxidase trafficking coincides with phagosome neutralization and supports depolymerization of DHN-melanin. (A) Representative images of D. discoideum expressing CybA-mCherry after 1 h of infection with FITC-stained conidia of the wild type to assess the phagosomal pH and NADPH oxidase activity simultaneously. The scale bar is 5 μm. (B) FITC-based pH measurements of phagosomes at 1 h p.i. Experiments were performed in 3 biological replicates, and statistically significant differences were calculated with a Bonferroni *post hoc* test after a two-way ANOVA. (C) FITC-based pH measurements of CybA-mCherry-positive phagosomes of infected cells at 2 h p.i. Experiments were performed in 3 biological replicates. Statistically significant differences were calculated with a *t* test. (D) H_2_O_2_-dependent and pH-dependent degradation of synthetic melanin derived from 1,8-DHN or melanin ghosts of wild-type conidia of A. fumigatus. H_2_O_2_ concentrations are given as wt/vol.

The NADPH oxidase (NOX) is a heteromultimeric, membrane-bound complex that produces intraphagosomal reactive oxygen species (ROS). The enzyme also plays an important role during A. fumigatus infection in humans (reviewed in reference 29). D. discoideum encodes three NOX catalytic subunits, i.e., *noxA* to *noxC*, with NoxA and NoxB being homologues of the mammalian gp91^phox^ subunit. A single gene, *cybA*, encodes the only D. discoideum homologue of the p22^phox^ subunit of the mammalian NADPH oxidase (20, 30). With wild-type conidia, CybA was detectable at the phagosome only after 1 h of infection, and primarily on near-neutral PLs, while highly acidic phagosomes were generally devoid of CybA (Fig. 5A). However, after 1 h postinfection (p.i.), the phagosomal pH was still highly variable among different phagosomes even within single cells (Fig. 5B), which may have been the result of asynchronous and repeated internalizations. At 2 h p.i., 81% of all phagosomes of the wild type were positive for ROS, whose production was at least partially dependent on NOX (Fig. S5). At that late time point, a proportion of CybA-positive phagosomes containing wild-type conidia displayed very high pH values between 8 or 9, averaging 7.2 (Fig. 5C). The pH in CybA-positive phagosomes containing the melanin-deficient *ΔpksP* conidia was only 6.2 on average. Both of these effectors, neutral pH and ROS, were sufficient to depolymerize DHN-melanin *in vitro* (Fig. 5D), suggesting the presence of DHN-melanin-derived degradation products in phagosomes at later stages of infection.

10.1128/mBio.00862-20.5 FIG S5 Imaging of ROS in D. discoideum during infection with resting conidia of the wild type. (A) Amoebae were incubated with fungal conidia for 1 h and stained with DHE. Phagosomes containing wild-type conidia were either positive (red arrow) or negative (white arrow) for DHE fluorescence. (B) DHE-based determination of levels of ROS-positive phagosomes in D. discoideum after infection with wild-type conidia. Experiments were performed in 3 biological replicates. Statistically significant differences were calculated with the *t* test. Download FIG S5, TIF file, 0.5 MB. Copyright © 2020 Ferling et al. 2020 Ferling et al. This content is distributed under the terms of the Creative Commons Attribution 4.0 International license.

### Lysosome fusion indicates damage to A. fumigatus-containing phagosomes.

**FIG 6:**
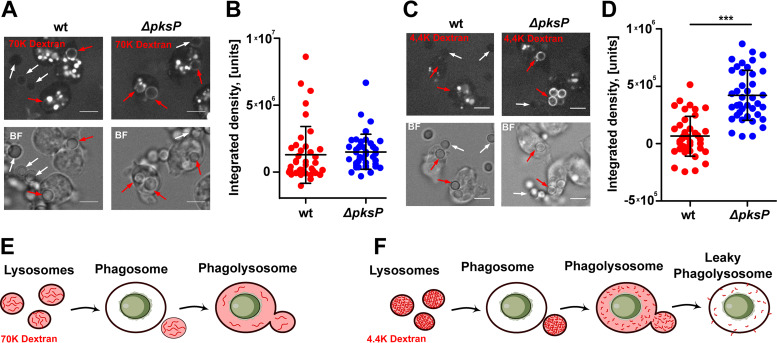
Dextran leakage and Vps32 trafficking to conidium-containing phagolysosomes. (A and C) Cells of D. discoideum were loaded with fluorescent dextran at a molecular weight of 70,000 Da (A) or 4,400 Da (C) and subsequently infected with A. fumigatus conidia. Images were captured after 300 min p.i. Internalized conidia and free conidia are indicated by red and white arrows, respectively. (B and D) Quantification of red fluorescence of the two dextrans (70 kDa [B] and 4.4 kDa [D]) as integrated densities in conidium-containing phagosomes after 300 min p.i. Values were normalized by background subtraction of free conidia. Data are based on results from 3 biological replicates, with statistically significant differences calculated in a one-way ANOVA with *P* values of <0.0001. (E and F) Schematic representation of size-discriminated leakage of dextran from phagolysosomes for 70-kDa dextran (E) and 4.4-kDa dextran (F).

Phagosome maturation involves the fusion of early/late phagosomes with lysosomes, which unload proteolytic enzymes for digestion of the phagolysosomal content. To monitor lysosomal fusion with phagosomes at later stages of infection, lysosomes of amoebae were loaded with fluorescently labeled 70-kDa dextran prior to infection with conidia. At 5 h p.i., dextran was visible as a ring in conidium-containing PLs (Fig. 6A). By measuring the normalized integrated density of these rings, we concluded that the phagosome-lysosome fusion was equally effective for melanized and nonmelanized conidia, although the results were highly variable due to repeated cycles of uptake and exocytosis (Fig. 6B). We substantiated these data by analyzing vacuolin, a postlysosomal marker that represents a functional homologue of the metazoan lipid-raft microdomain organizer flotillin (31). In agreement with the data obtained for dextran accumulation, vacuolin gradually accumulated in the membrane of both PLs containing either wild-type or *ΔpksP* conidia (Fig. S6). Collectively, these data suggested that lysosomal fusion was not inhibited.

10.1128/mBio.00862-20.6 FIG S6 VacB trafficking on infected phagosomes. (a) Representative images of VacB-GFP-expressing cells infected with conidia of the wild type (wt) or the Δ*pksP* strain at 2 h p.i. White arrows indicate conidium-containing phagosomes and colocalization of VacB. Scale bars are 5 μm. (b) Quantification of the integrated density for VacB-GFP at conidium-containing phagosomes. Download FIG S6, TIF file, 0.7 MB. Copyright © 2020 Ferling et al. 2020 Ferling et al. This content is distributed under the terms of the Creative Commons Attribution 4.0 International license.

To follow the integrity of the phagolysosomal membrane, we preloaded lysosomes of the amoebae with a dextran of low molecular mass (4.4 kDa). In contrast to the results seen with the larger dextran, amoebae infected with wild-type conidia displayed almost no rings whereas even the smaller dextran was fully retained in PLs containing the melanin-deficient mutant (Fig. 6C and D). These results suggested that wild-type conidia, after prolonged intracellular transit, resided in leaky PLs, in contrast to the conidia of the *ΔpksP* strain (Fig. 6E and F).

### Phagolyosomes with wild-type conidia do not trigger recruitment of the ESCRT repair machinery.

**FIG 7:**
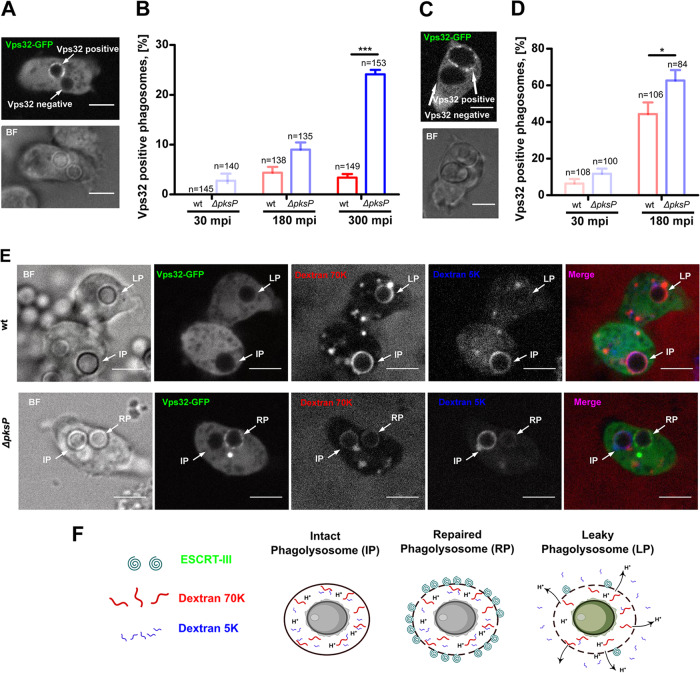
Vps32 localization to damaged phagolysosomes. (A and C) Representative images of Vps32-GFP-expressing cells infected with dormant conidia (A) or swollen conidia (C) at an MOI of 10 after 180 min p.i. (B and D) Quantification of Vps32-GFP localization to phagosomes containing resting conidia (B) or swollen conidia (D). Statistically significant differences were calculated with a Bonferroni *post hoc* test after a two-way ANOVA, with asterisks indicating significance (*, *P* < 0.05; **, *P* < 0.01; ***, *P* < 0.001). Scale bars are 5 μm. Vps32 was absent from damaged phagolysosomes containing DHN-melanized conidia. (E) Vps32 localization and phagolysosome integrity in single cells. Cells of D. discoideum expressing Vps32-GFP were first loaded with RITC-dextran of 70,000 Da and blue dextran of 5,000 Da and were subsequently infected with dormant conidia of the wild-type strain or Δ*pksP* mutant. Images were captured at 180 min p.i. Scale bars are 5 μm. IP, intact phagolysosome; LP, leaky phagolysosome; RP, repaired phagolysosome. (F) Schematic illustration of the experimental results.

Recently, it was demonstrated that disruptions of endolysosomes can be repaired by the endosomal sorting complex required for transport (ESCRT) machinery (32, 33). In D. discoideum, Vps32 is a homologue of the CHP4A, CHP4B, and CHP4C proteins of the ESCRT-III complex in metazoa. The protein localizes to injuries at the plasma membrane and endomembranes (21). We hypothesized that phagolysosomal damage due to long-lasting and repeated conidial infections would eventually trigger the recruitment of this complex, which would be measurable by the use of a Vps32-GFP-expressing cell line (Fig. 7). Infection of this cell line with conidia of the *ΔpksP* strain triggered the recruitment of the ESCRT-III machinery to phagolysomes in a time-dependent manner, with a maximum of 25% of Vps32-positive PLs after a long-term confrontation of 5 h (Fig. 7A and B). From the dextran leakage experiments, which indicated more damage to PLs containing the wild type, we would have expected an even more robust recruitment of Vps32 for melanized conidia. Unexpectedly, Vps32 was recruited only to less than 5% of wild-type conidium-containing PLs over the entire period. We reasoned that any onset of intracellular swelling or germination of the conidium would trigger a robust damage response. As expected, preswollen conidia, which had lost their melanin coat, recruited higher levels of the Vps32 protein to the phagosome (Fig. 7C and D). Here, the numbers for Vps32-positive phagosomes exceeded 40 and 60% for the wild type and the mutant, respectively. This indicates that leakage from wild-type-containing PLs may be due to the inhibition of recruitment of the ESCRT repair machinery.

We further substantiated the lack of ESCRT recruitment to damaged, wild-type conidium-containing phagosomes by combining all three reporters; i.e., Vps32-expressing cells preloaded with dextrans of both molecular masses, but differentially labeled, were infected with either wild-type or *ΔpksP* conidia (Fig. 7E and F). Infections with the wild type led to leaky phagosomes which were positive only for the 70-kDa dextran and devoid of both the 5-kDa dextran and Vps32. Some phagosomal damage was also detected with *ΔpksP* conidia, visualized by a partial loss of the 5-kDa dextran. However, Vps32 was recruited to these phagosomes, indicating active repair. This result was further supported by the observation that the smaller dextran was well retained in such Vps32-positive phagosomes (Fig. 7E; see the panel for 5-kDa dextran).

### Delayed formation of autophagosomes with DHN-melanized conidia.

D. discoideum exploits the autophagy pathway to enclose intracellular pathogens in a membrane-sealed phagophore (20). To test whether a conidial infection would also trigger formation of autophagsomes, we used D. discoideum cells expressing GFP-Atg8, the amoeba homolog of the mammalian protein LC3 and a major marker for autophagy (34).

**FIG 8:**
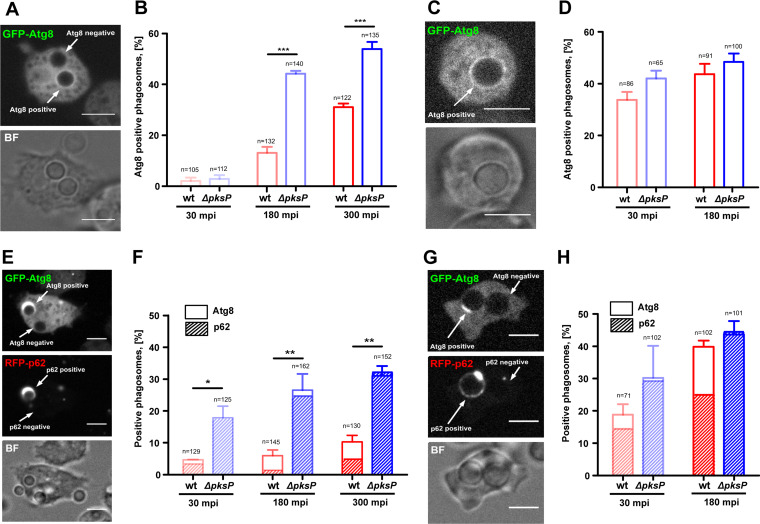
Autophagy activation in response to candida of A. fumigatus. (A, C, E, and G) Representative images of D. discoideum cells expressing GFP-Atg8 alone (A and C) and together with p62-RFP (E and G) during infection with resting (A and E) or swollen (C and G) conidia of the A. fumigatus wild type. (B, D, F, and H) Quantification of recruitment of GFP-Atg8 alone (B and D) and GFP-Atg8 together with p62-RFP (F and H) to phagosomes infected with resting (B and F) or swollen (D and H) conidia of the A. fumigatus wild-type strain or *Δpksp* mutant. Experiments were performed in 3 biological replicates. Statistically significant differences were calculated with a Bonferroni *post hoc* test after a two-way ANOVA (***, *P* < 0.001). MOI = 10. Scale bar = 5 μm.

GFP-Atg8 was recruited gradually during conidial infection, and approximately half of the phagosomes containing melanin-deficient conidia were positive for GFP-Atg8 at 180 min p.i. (Fig. 8A and B). At that time point, only 10% of the phagosomes with melanized conidia had recruited Atg8, indicating that the presence of DHN-melanin could repress autophagy. To further verify that the autophagy repression is attributable to DHN-melanin, conidia of both strains were preswollen for 5 h, leading to a loss of melanin in the wild type. More than 30% of the amoebae infected with swollen conidia recruited Atg8 within the first 30 min of infection, and no significant differences were seen regardless of whether the cells were confronted with wild-type conidia or *ΔpksP* conidia (Fig. 8C and D). Since the LC3 protein in metazoa is involved in canonical autophagy and LAP, we aimed to clarify whether canonical autophagy or LAP was activated by infection with A. fumigatus. Results from a reporter cell line that simultaneously expressed GFP-Atg8 and the canonical autophagy adaptor RFP-p62 (Fig. 8E to H) confirmed that canonical autophagy rather than LAP was activated in the amoebae. However, phagosomes infected with DHN-melanin-covered conidia showed delays in the formation of autophagosomes, which may further substantiate membrane damage.

## DISCUSSION

A. fumigatus infections are usually acquired from environmental sources, suggesting that virulence determinants could also serve a protective role outside the human host. We have used two amoeba models to follow the antagonistic interaction of A. fumigatus conidia with amoebae in real time. Conidia covered with the green pigment DHN-melanin had higher rates of survival when confronted with the phagocytic fungal predator P. aurantium, resulting primarily from far lower rates of internalization. A similar trend of a reduced uptake was also seen for the nonfungivorous amoeba D. discoideum. Resting, wild-type conidia were rarely ingested, despite high levels of initial attachment. The absence of the fungal pigment generally triggered higher phagocytic ratios in a manner similar to the results obtained for human macrophages. For the latter, DHN-melanin of A. fumigatus is well known to interfere with phagocytosis rates (reviewed in reference 6). Such parallels might indicate that DHN-melanin serves as a pigment protective against a wide range of phagocytic cells, which may either belong to the innate immune defense of metazoa or be distant members within the highly diverse kingdom of amoebozoa.

The initial maturation step of acidification in the amoeba upon infection with a conidium of A. fumigatus was only marginally affected. This result differs from the findings reported for murine and human macrophages where the acidification of phagosomes was delayed by melanized conidia (4). The acidification defect has recently been attributed to the interference of DHN-melanin with the lipid rafts that are required for v-ATPase assembly (11). Differences observed in the two models imply that the presence of DHN-melanin may result in differential targeting of animal and protozoal phagocytic cells. Another possible reason for the difference in acidification of A. fumigatus-containing phagosome in amoeba and macrophages may be general dissimilarities in the phagosome maturation process. For example, in D. discoideum, acidification and V-ATPase delivery occur within minutes after closure of the phagocytic cup (27). A multitude of roles have been attributed to ROS during phagosome maturation in innate immune cells and amoebae. Although fluorescence-based detection of the proteins may result in underestimation of their actual dynamics, our results indicate that NADPH oxidase assembly and ROS production coincide with the disassembly of the V-ATPase and neutralization of the phagosome. Such a neutralizing function of phagosomal ROS has been repeatedly reported for dendritic cells (35, 36). One possible explanation for the reduced neutralization kinetics seen with wild-type conidia might be that DHN-melanin initially quenches ROS. Our results on the degradative role of ROS in synthetic and fungal DHN-melanin support this idea.

Detailed single-cell phagosome maturation analyses revealed that DHN-melanin-positive conidia showed delayed phagosome maturation and intracellular transit. We further demonstrated that these phagolysosomes containing conidia with DHN-melanin were leaky. The fact that a low pH was maintained throughout the first 90 min suggests that membrane damage occurs only at later stages of the infection, presumably after or even as a direct result of ROS production in the PL. Membrane damage to PLs has also been reported for the yeasts C. neoformans and C. albicans, resulting most likely from mechanical forces during intracellular replication and hyphal expansion, respectively (37–39). In D. discoideum, membrane integrity may be weakened due to the intrinsic production of ROS and the onset of conidial swelling and may be further enhanced by fungal mycotoxins, such as the spore-borne polyketide trypacidin (17). In response, D. discoideum recruits the ESCRT machinery to damaged intracellular membranes, as observed for Mycobacterium marinum-containing vacuoles (21, 40). ESCRT was recruited most effectively to PLs of swollen conidia and to those containing resting, melanin-free conidia. However, leakage was primarily observed for PLs with the wild type, and we thus conclude that DHN-melanin or its degradation products interfere with the recruitment of the ESCRT machinery. How ESCRT-III is recruited in D. discoideum is currently not known. In mammalian cells, ESCRT-III recruitment to damaged plasma membranes and lysosomes was hypothesized to depend on the recognition of a local increase in levels of Ca^2+^ by ALIX and/or ALG2 (32, 33). In D. discoideum, ESCRT-III recruitment to sites of membrane damage strongly depends on the ESCRT-I component Tsg101 but appeared to be independent of Ca^2+^ (21). However, the possibility cannot be excluded that chelation of Ca^2+^ by DHN-melanin, as observed in phagosomes of innate immune cells (41), may also attenuate ESCRT-III recruitment in D. discoideum.

In amoebae, autophagy serves as another defense line against infection (20). Our results demonstrate that D. discoideum activates autophagy to combat an infection with conidia of A. fumigatus. DHN-melanin or its degradation products suppressed full activation of canonical autophagy, presumably through the inhibition of adapter marker p62 assembly to the phagosome. Delayed recruitment of the autophagy machinery may further substantiate the intracellular damage and thus prolong intracellular transit. Interestingly, the noncanonical LC3-associated phagocytosis (LAP) process promotes killing of A. fumigatus by human macrophages but is blocked through Ca^2+^ sequestration by DHN-melanin (9, 41). Atg8 is the amoeba orthologue of mammalian LC3 (42), and thus, LAP may be a relatively new evolutionary adaptation in higher eukaryotes that helps them to combat the conidia as a pathogen.

How DHN-melanin or its degradation products suppress the cell autonomous repair machinery of the amoeba remains unclear. Swelling and germination are known to be initiated inside the PLs of certain types of macrophages and amoebae, including D. discoideum (15, 16, 43). During these processes, DHN-melanin was shed from the conidial surface and, at least in *in vitro*, synthetic DHN-melanin and melanin ghosts were more efficiently degraded by H_2_O_2_ at neutral pH, indicating that unknown degradation products of DHN-melanin may be present inside the phagolysosome. Both a neutral-to-alkaline pH and the presence of ROS have long been known to be key mediators during the biochemical breakdown of chemically diverse melanins (44, 45). The finding that low micromolar levels of 1,8-DHN can fully inhibit the motility of D. discoideum indicates that DHN-melanin or DHN derivatives can exert toxic effects on the host cell. Mammals can exploit a specific C-type lectin as a DHN-melanin sensor. This receptor (MelLec) has recently been found to play an essential role in human antifungal immunity (3). There is no record of Amoebozoa featuring structurally similar C-type lectins, but they may exploit convergent, yet-to-be-identified orthologues.

It is likely that damage to the sealed phagolysosome would lead to an influx of nutrients and would help the fungus to establish a germination niche. Although this advantage may be restricted to nonspecialized phagocytes that are unable to kill the fungus, we also found a protective role for DHN-melanin in encountering a fungivorous amoeba, demonstrating that surface exposure of DHN-melanin provides an overall selective advantage in phagocytic predator-prey interactions in environmental reservoirs.

## MATERIALS AND METHODS

### Strain and culture conditions.

D. discoideum cells were axenically grown in petri dishes (Greiner Bio-One, Austria) (94 mm) in HL5 medium (Formedium, United Kingdom) supplemented with 1% (wt/vol) glucose and penicillin-streptomycin (Genaxxon Bioscience, Germany) at 10 U ml^−1^ of penicillin and 10 μg ml^−1^ of streptomycin and were split every 2 to 3 days before reaching confluence. Protostelium aurantium var. *fungivorum* (46) was grown in phosphate-buffered saline (PBS) (pH 6.6) with Rhodotorula mucilaginosa as a food source at 22°C. Aspergillus fumigatus strains were grown in Aspergillus minimal medium (AMM) or Czapek Dox medium (CZD; Thermo Fisher Scientific, Germany) at 37°C, supplemented with 1.5% (wt/vol) agar for growth on solid media.

10.1128/mBio.00862-20.6 TABLE S1 Strains used in the study. Download Table S1, XLSX file, 0.02 MB. Copyright © 2020 Ferling et al. 2020 Ferling et al. This content is distributed under the terms of the Creative Commons Attribution 4.0 International license.

### Quantification of phagocytosis and conidial attachment to phagocytes.

P. aurantium or D. discoideum cells with events of attachment to or phagocytosis of A. fumigatus conidia, silica beads, and melanin ghosts were quantified from microscopic images captured at 1 h p.i. Phagocytic ratios and uptake ratios were calculated based on *n* = 100 amoeba trophozoites for each experiment. Statistically significant differences were calculated with a Bonferroni *post hoc* test after a two-way analysis of variance (ANOVA).

### Resazurin-based survival assay after amoeba predation.

A total of 1 × 10^6^ conidia of A. fumigatus were placed in 96-well tissue culture in 100 μl CZD media. Conidia were confronted with P. aurantium directly (dormant conidia) or after preincubation at 37°C for 6 h (swollen) at an MOI of 10. P. aurantium was collected from precultures on R. mucilaginosa. The liquid medium was aspirated from the plate and washed two times with PBS to remove residual yeast cells. Trophozoites were added at prey/predator ratios of 10:1 were and incubated at 22°C for 18 h. The plate was then transferred to 37°C for 1 h in order to kill the amoeba and induce fungal growth. Resazurin (catalog no. R7017; Sigma-Aldrich, Taufkirchen, Germany) (0.002% [wt/vol]) was added to quantify the amount of fungal growth to each well in real time as the level of fluorescence, measured in intervals of 30 min over 80 h at an excitation wavelength (λ_ex_) of 532 nm and an emission wavelength (λ_em_) of 582 nm using an Infinite M200 Pro fluorescence plate reader (Tecan, Männedorf, Switzerland).

### Microscopy and image analysis.

Microscopy was carried out on an Axio Observer spinning disc confocal microscope (Zeiss) using ZEN Software 2.6. Fluorescent stains and proteins were excited using 488-nm and 561-nm laser lines. Quantification of fluorescence intensity was performed using ImageJ (https://imagej.nih.gov).

### Motility measurements with D. discoideum.

Single D. discoideum cells were tracked for 10 min by microscopy, and the velocity of the cells was measured by manual tracking with the TrackMate plugin of ImageJ. All experiments were performed with three biological replicates with at least 10 cells measured per replica.

### Measurement of phagosome acidification.

Conidia of the wild type and the *ΔpksP* strain were stained with FITC and CF594 fluorophores for 10 min and washed two times with PBS. First, an FITC-fluorescence-based pH calibration curve was generated by imaging the labeled conidia in HL5 buffered to pH values from 3.5 to 8.0. The integrated density of FITC fluorescence was measured for at least 10 conidia with ImageJ, and the log values of the results were plotted against the pH to generate a calibration curve. To determine the intraphagosomal pH, D. discoideum cells at concentrations of 10^6^ ml^−1^ were axenically grown as an adherent culture in ibidi dishes (ibidi, Germany) in a total volume of 2 ml HL5 supplemented with 1% (wt/vol) glucose. To synchronize the D. discoideum cells, plates were cooled to 4°C for 10 min on an ice-cold metal plate before addition of conidia. Amoebae were confronted with conidia at an MOI of 10 and briefly centrifuged (500 × *g*, 2 min). Excess medium was aspirated, and a 1% (wt/vol) agarose sheet (1.5 by 1.5 cm) was placed on top of the cells. Cells were imaged at 3-min-to-1-min frame intervals, for up to 4 h, with a spinning disc confocal system (Axio Observer with a Cell Observer spinning disc unit; Zeiss) using the 63× oil lens objective. Image processing and quantification of fluorescence intensity were performed with ImageJ. GraphPad5 Prism software was used to plot graphs and perform statistical analyses.

### Construction of a CybA reporter strain of D. discoideum.

To express CybA fused to the N terminus of mCherry, *cybA* was PCR amplified from D. discoideum AX2 (Gerisch) genomic DNA (gDNA) with primers jd102for (5′-GCGGATCCAAAAATATGGGAAAATTTAAACTTGGT-3′), which contains a BamHI site and a Kozak sequence in front of the first ATG (start codon), and jd102rev (5′-GCACTAGTATCCTGTTTATCGAAAAAATCACCT-3′), which contains a SpeI site. The PCR product was cloned into pJET2.1 using a CloneJET PCR cloning kit (Thermo Fisher Scientific). Subsequent to confirmation by sequencing, the *cybA* insertion was excised with BamHI and SpeI, gel purified, and cloned into plasmid pDM1097 (kindly provided by Douwe Veltman), which had been cut with the same enzymes and treated with alkaline phosphatase, to produce pDM1097-cybA, which encodes the CybA-mCherry fusion protein. The plasmid was electroporated into D. discoideum AX2 (Gerisch), and transformed amoebae were selected with G418 at a final concentration of 5 μg ml^−1^.

### Imaging of phagosomal reporter proteins.

D. discoideum cell lines expressing Vps32-GFP, GFP-Atg8 or GFP-Atg8/RFP-p62, or CybA-mCherry were seeded in 300 μl of HL5 supplemented with 1% (wt/vol) glucose at a cell density of 10^5^ ml^−1^ in 8-well microscopy dishes (ibidi, Germany) and left for 2 h to adhere. Fungal conidia were added to the wells at an MOI of 10, and the dishes were centrifuged for 30 s at 500 × *g*. Tile scans of selected areas at given time points were performed with a spinning disc confocal system (Axio Observer with a Cell Observer spinning disc unit; Zeiss) using the 63 × oil lens objective. Each experiment was performed in three biological replicates. Fluorescent cells containing conidia were considered for quantification with ImageJ. GraphPad5 Prism software was used to perform statistical tests and to plot graphs.

### Visualization of ROS generation in D. discoideum.

Amoebae were infected in 8-well Ibidi dishes with resting conidia of A. fumigatus at an MOI of 10. After 2 h of coincubation, the superoxide indicator dihydroethidium (DHE; Thermo Fisher Scientific) was added to the wells to reach a final concentration of 10 μM. After 10 min, the sample was imaged with a red laser and a blue laser. Experiments were performed in three biological replicates.

### Coincubation with dextran.

D. discoideum cells were incubated with dextran at a molecular weight (MW) of 70,000 (labeled with rhodamine-B-isothiocyanate [RITC]; catalog no. R9379, Sigma-Aldrich) or with dextran at an MW of 4,400 (labeled with tetramethyl rhodamine isocyanate [TRITC]) (catalog no. T1037, Sigma-Aldrich) and with blue dextran at an MW of 5,000 (catalog no. 90008; Sigma-Aldrich). Final concentrations were 0.5 mg ml^−1^ (70-kDa dextran) and 1.5 mg ml^−1^ (4.4-kDa and 5-kDa dextran). After 2 h, the cells were washed with fresh media to remove extracellular dextran and were infected with fungal conidia. Dextran-loaded lysosomes fused with conidium-containing phagosomes, thus creating fluorescent rings around the ingested conidia. Damage to the plasma membrane was visible due to selective diffusion of the smaller dextrans into the cytosol.

### Computational modeling.

A Monte Carlo computational model was used to assess the population-wide distribution of acidic phagosomes during infections with conidia of the wild-type strain and the *ΔpksP* mutant. Statistically significant differences were calculated with a Bonferroni *post hoc* test after two-way ANOVA (*P* < 0.0001). This simulation performs a risk analysis by building models that integrate the range of values obtained in previous experiments for each fungal cell line (e.g., phagocytosis rate, average time of conidia inside acidified phagosome, time point of exocytosis). It then repeatedly executes the calculation, each time using a different set of random values from the probability functions. The generated simulations predict possible outcomes of the infection for the whole amoeba population for each fungal cell line. The simulation code is available online at https://github.com/devlxf/FungiSim.

### Synthetic polymerization of 1,8-DHN-melanin.

Melanin ghosts were prepared as previously described (47) using a concentration of 10^9^ particles ml^−1^. Synthetic melanin was polymerized spontaneously from 1,8-dihydroxynaphthalene (Sigma) over 3 days in phosphate buffer in a 48-well plate. H_2_O_2_ was added in various concentrations to the wells, and images were captured after 48 h.
